# Antigen Remodeling in Colorectal Cancer: How Radiotherapy and Chemotherapy Enhance Immunotherapy Responsiveness

**DOI:** 10.3390/cancers18040715

**Published:** 2026-02-23

**Authors:** Yuki Matsumi, Kunitoshi Shigeyasu, Toshiaki Takahashi, Kazuya Moriwake, Masashi Kayano, Toshiyoshi Fujiwara

**Affiliations:** Department of Gastroenterological Surgery, Okayama University Graduate School of Medicine, Dentistry, and Pharmaceutical Sciences, Okayama 700-8558, Japan; par65zf9@okayama-u.ac.jp (Y.M.); pnyn10i9@s.okayama-u.ac.jp (T.T.); pci547kp@s.okayama-u.ac.jp (K.M.); p4vn6kup@s.okayama-u.ac.jp (M.K.); toshi_f@md.okayama-u.ac.jp (T.F.)

**Keywords:** colorectal cancer, immunotherapy, radiotherapy, chemotherapy, neoantigens

## Abstract

Colorectal cancer (CRC) is traditionally considered a “cold tumor” characterized by low immunogenicity and limited responsiveness to immune checkpoint inhibitors (ICIs). However, recent findings reveal that cytotoxic modalities can reprogram this immunologically inert landscape. This review integrates these evolving concepts to guide the optimization of future treatments. Radiotherapy induces extensive DNA double-strand breaks, which may generate de novo mutations through error-prone repair while simultaneously exposing cryptic antigens via increased transcriptional instability, alternative splicing, and enhanced proteasomal processing. Chemoradiation also amplifies epigenetic and epitranscriptomic sources of neoepitope diversity, including RNA editing and stress-induced splicing alterations, expanding the immunopeptidome beyond canonical mutation-driven neoantigens. These changes collectively enhance antigen presentation and facilitate T-cell priming. Chemotherapy further reduces immunosuppressive cell populations and promotes dendritic cell activation, creating a permissive milieu for subsequent immune engagement. Clinically, the VOLTAGE studies demonstrated that long-course chemoradiotherapy can sensitize even mismatch repair–proficient rectal cancers to PD-1 blockade, yielding clinically meaningful pathological responses. In contrast, mismatch repair–deficient rectal tumors may respond completely to ICIs alone. Short-course radiotherapy combined with chemotherapy and ICIs has also shown encouraging activity in the setting of total neoadjuvant therapy. Collectively, these findings support a paradigm in which radiotherapy, chemotherapy, and epigenetic/epitranscriptomic alterations—including RNA editing—act as potent modulators of tumor antigenicity. By expanding the neoantigen repertoire and reshaping the tumor microenvironment, these strategies can transform CRC from a cold tumor into one that is increasingly responsive to immunotherapy.

## Simple Summary

Colorectal cancer is often considered a “cold tumor” that responds poorly to immunotherapy. However, tumor antigenicity is not static and can be reshaped by cancer treatments. This review summarizes emerging evidence that chemoradiotherapy and chemotherapy can remodel tumor antigens by inducing DNA damage, transcriptional stress, and epigenetic/epitranscriptomic alterations, including RNA editing. These therapy-induced changes may generate new or unmasked neoepitopes, thereby enhancing immune recognition even in microsatellite-stable colorectal cancer. By integrating mechanistic insights with recent clinical trial data, we highlight how combining cytotoxic therapies with immune checkpoint inhibitors may overcome immune resistance. Understanding therapy-driven antigen remodeling provides a framework for optimizing immunotherapy strategies and identifying biomarkers of response in colorectal cancer.

## 1. Introduction

Colorectal cancer (CRC) remains a leading cause of cancer-related mortality worldwide and is biologically characterized as a prototypical “cold tumor” [1]. Most colorectal cancers (CRCs), particularly mismatch repair–proficient (pMMR or MSS) tumors, exhibit low inherent immunogenicity. These tumors typically show sparse effector T-cell infiltration, limited antigenicity, and an immunosuppressive tumor microenvironment [1,2]. These features contribute to CRC’s generally poor response to immune checkpoint inhibitors (ICIs). In contrast, mismatch repair–deficient (dMMR/MSI-H) tumors demonstrate significant sensitivity to ICIs [3,4]. Consequently, immunotherapy alone has yielded only modest clinical benefit for most patients with CRC, underscoring the need for strategies that can actively modulate the tumor immune landscape.

Emerging evidence from both translational research and clinical trials indicates that the “cold” status of CRC is not fixed; instead, its immunogenicity can be reshaped by specific therapeutic pressures. Cytotoxic treatments such as radiotherapy and chemotherapy not only have direct tumoricidal effects but also induce significant biological changes that enhance antigen presentation and promote antitumor immunity. Radiotherapy induces DNA double-strand breaks and transcriptional perturbations, leading to de novo and cryptic antigens [5]. Chemotherapy can induce immunogenic cell death and modulate immunosuppressive cell populations [6]. Moreover, epigenetic and epitranscriptomic mechanisms—including RNA editing, alternative splicing, and other stress-induced transcriptomic changes—expand the diversity of potential neoepitopes, effectively remodeling the antigenic landscape of CRC [7,8].

This review integrates current mechanistic insights and clinical evidence to elucidate how radiotherapy, chemotherapy, and epigenetic/epitranscriptomic alterations converge to restore antigenicity and sensitize CRC to immunotherapy. By examining these processes through the unifying concept of antigen remodeling, we outline a framework that may transform therapeutic strategies for a disease historically refractory to immunotherapy.

## 2. Methods

This narrative review was based on a structured literature search of PubMed and Web of Science databases through January 2026. Search terms included combinations of “colorectal cancer,” “radiotherapy,” “chemoradiotherapy,” “immunotherapy,” “neoantigen,” “RNA editing,” “epigenetic,” “epitranscriptomic,” and “immune microenvironment.” Both preclinical and clinical studies were considered. When conflicting data were identified, emphasis was placed on reproducible findings and studies with clinical relevance.”

## 3. Immunobiology of Colorectal Cancer as a Cold Tumor

CRC is widely recognized as a prototypical “cold” tumor, characterized by low baseline immunogenicity and limited spontaneous antitumor immune activity [9]. This phenotype is largely driven by the composition and spatial organization of the tumor immune microenvironment. Many CRCs exhibit an immune-desert phenotype, in which effector T cells are scarce within the tumor parenchyma, or an immune-excluded profile, in which T cells accumulate at the invasive margin but fail to infiltrate tumor nests. Both patterns result in insufficient cytotoxic T-cell engagement and limited responsiveness to immune checkpoint inhibition [9].

The inherently low tumor mutational burden (TMB) of most CRCs contributes to their weak antigenicity. While tumors with high TMB generate a broad repertoire of neoepitopes that can elicit robust T-cell responses, most CRCs—particularly mismatch repair–proficient (pMMR or MSS) tumors—harbor relatively few immunogenic neoantigens [10]. In contrast, mismatch repair–deficient (dMMR/MSI-H) CRCs exhibit significantly high mutational burdens and dense lymphocytic infiltration, a combination that underlies their exceptional sensitivity to PD-1 blockade [11]. This sharp dichotomy between MMR-proficient and MMR-deficient CRC underscores the importance of antigen quantity and quality in determining responsiveness to immunotherapy.

In addition to antigenicity, CRC harbors multilayered mechanisms of resistance to immunotherapy. These include insufficient T-cell infiltration, low or heterogeneous PD-L1 expression, and a stromal compartment enriched in cancer-associated fibroblasts alongside TGF-β–driven signaling that reinforces immune exclusion [12,13]. Additional barriers, such as myeloid-derived suppressor cells and regulatory T cells, further suppress antitumor immunity, creating a microenvironment that is structurally and functionally resistant to immune activation [10]. Several genetic and immunological impediments may contribute to treatment resistance in a subset of patients. These include defects in antigen presentation machinery (e.g., HLA loss or B2M alterations), sustained immunosuppressive populations such as regulatory T cells and myeloid-derived suppressor cells, and stromal barriers driven by TGF-β signaling. Such factors may limit effective T-cell infiltration and function despite therapeutic efforts to remodel antigens.

Collectively, these features characterize CRC as an immune-refractory malignancy in its untreated state. However, they also underscore the potential for therapeutic strategies that can alter antigenicity and reshape the immune microenvironment, paving the way for the concept of antigen remodeling explored in subsequent sections.

**Key Take-Home Point:** Most MSS colorectal cancers remain immunologically cold due to limited antigenicity, impaired antigen presentation, and stromal or myeloid-mediated immune exclusion.

## 4. Antigen Remodeling: Concept and Mechanistic Framework

Tumor antigenicity—the ability of tumor-derived peptides to be recognized by the host immune system—is a key determinant of response to immunotherapy [14]. This antigenic landscape is defined not only by the quantity and quality of neoepitopes derived from genetic mutations but also by a broader immunopeptidome resulting from alternative splicing, aberrant translation, posttranscriptional modifications, and epigenetic/epitranscriptomic regulation [14,15]. In CRC, where baseline tumor mutational burden is typically low, the limited diversity of immunogenic peptides contributes to its classification as an immunologically cold tumor [16]. Consequently, mechanisms that expand or diversify the antigen repertoire are critical for improving sensitivity to immunotherapy.

Antigen remodeling refers to the dynamic reconfiguration of tumor antigenicity through therapeutic or stress-induced alterations at the DNA, RNA, and epigenetic/epitranscriptomic levels. Cytotoxic treatments—especially radiotherapy—act as potent drivers of this process. Ionizing radiation induces numerous DNA double-strand breaks, and subsequent error-prone repair can generate de novo mutations, including frameshifts that give rise to neoantigens [5]. Concurrently, radiation disrupts transcriptional fidelity and increases alternative splicing events, thereby exposing cryptic antigens that were previously untranscribed, untranslated, or insufficiently processed for MHC class I presentation [5]. Chemotherapy and other cellular stressors further amplify this phenomenon by promoting immunogenic cell death, reorganizing proteasomal processing, and activating inflammatory signaling pathways that facilitate enhanced antigen presentation [6].

Beyond genetic alterations, epigenetic and epitranscriptomic mechanisms—including RNA editing, splicing dysregulation, shifts in RNA degradation pathways, and changes in chromatin accessibility—contribute additional layers of antigen diversification [8]. These processes can generate noncanonical peptides independent of DNA-level mutations, expanding the immunopeptidome in a manner particularly relevant for tumors with low mutational burden, such as mismatch repair–proficient CRC. Collectively, these nuclear and epitranscriptomic perturbations provide a mechanistic basis for shifting CRC from an antigen-poor, immune-refractory state to one that is increasingly recognized by cytotoxic T cells.

This concept of antigen remodeling differs fundamentally from traditional cancer immunoediting, which characterizes the immune-mediated selection of preexisting tumor subclones. Conversely, antigen remodeling emphasizes therapy-induced diversification, whereby cytotoxic stress generates novel antigenic features rather than merely influencing the fitness of existing clones [17]. By reconceptualizing therapeutic interventions as active modulators of antigenicity, this framework offers a unifying rationale for integrating radiotherapy, chemotherapy, and epigenetic/epitranscriptomic modulation to render cold CRC responsive to immunotherapy.

Table 1 summarizes the major therapy-induced pathways contributing to genetic and epigenetic/epitranscriptomic neoantigen diversification.

**Key Take-Home Point:** Antigen remodeling reflects therapy-induced diversification of tumor antigens through genetic and epitranscriptomic mechanisms, extending beyond static mutation burden.

## 5. Radiotherapy as an Antigen Generator

Radiotherapy (RT) has traditionally been viewed as a local cytotoxic therapy, yet accumulating evidence demonstrates that ionizing radiation profoundly reshapes tumor antigenicity, acting as an upstream driver of antigen remodeling. Radiotherapy may generate novel neoepitopes through DNA damage and transcriptional stress; however, direct evidence demonstrating T-cell recognition of therapy-induced neoepitopes in colorectal cancer patients remains limited [26]. Importantly, antigen diversification should be distinguished from broader immune activation processes, including enhanced trafficking, depletion of suppressor cells, or cytokine-mediated modulation of the tumor microenvironment.

A critical biological effect of RT is the induction of extensive DNA double-strand breaks (DSBs) [5]. Subsequent reliance on error-prone repair pathways—primarily nonhomologous end joining—leads to insertions, deletions, and frameshift mutations that generate de novo neoantigens. These newly formed peptides can diversify the immunopeptidome and serve as targets for T-cell recognition, even in tumors with inherently low mutational burden such as mismatch repair–proficient colorectal cancer [27].

In addition to direct genomic alterations, radiation disrupts transcriptional and posttranscriptional fidelity. Increased transcriptional noise, aberrant alternative splicing, and stress-induced transcript isoforms expose previously unexpressed or unstable peptides, leading to the emergence of cryptic antigens not present in the steady-state proteome. Concurrently, radiation modifies proteasomal processing and increases peptide turnover, further broadening antigen presentation.

At the cell surface, RT upregulates MHC class I expression, driven in part by interferon signaling and increased peptide availability [5]. This increased antigen-presenting capacity enhances the visibility of irradiated tumor cells to cytotoxic T lymphocytes. Central to this process is the activation of the cGAS–STING pathway, triggered by cytosolic DNA fragments generated during radiation-induced genomic stress [20,28]. STING activation leads to robust type I interferon (IFN-I) production, which enhances dendritic cell maturation, cross-priming, and the recruitment of effector T cells into the tumor microenvironment.

Clinically, these molecular effects manifest as increased CD8^+^ T-cell infiltration and adaptive PD-L1 upregulation in irradiated tumors—hallmarks of an “inflamed” immune phenotype. These shifts have been observed in rectal cancer following chemoradiotherapy and correlate with improved responses to PD-1 blockade in trials such as VOLTAGE. Collectively, these mechanisms position radiotherapy not merely as a means of direct tumor debulking but as a potent antigen generator capable of converting CRC from a cold tumor into one primed for immunotherapeutic engagement [29].

**Key Take-Home Point:** Radiotherapy may enhance immune responsiveness by inducing DNA damage-associated neoantigens and activating innate immune sensing pathways such as cGAS–STING.

## 6. Chemotherapy-Induced Immunomodulation

Although chemotherapy has long been viewed primarily as a cytotoxic strategy for tumor control, it is now evident that many chemotherapeutic agents exert profound immunomodulatory effects that complement antigen remodeling and enhance CRC responsiveness to immunotherapy. One of the most prominent mechanisms is the induction of immunogenic cell death (ICD) [6]. Agents such as oxaliplatin and certain fluoropyrimidine-based regimens induce endoplasmic reticulum stress and regulated necrosis, leading to the release of danger-associated molecular patterns, including calreticulin, ATP, and HMGB1. Oxaliplatin has been associated with immunogenic cell death primarily in preclinical studies, 5-fluorouracil may reduce myeloid-derived suppressor cells based on experimental and correlative clinical data, and irinotecan appears to exert predominantly cytotoxic effects with less clearly defined immune-stimulatory activity. These signals promote dendritic cell recruitment, antigen uptake, and the cross-priming of tumor-specific CD8^+^ T cells, effectively bridging cytotoxic injury and adaptive immune activation [6,22,30].

Chemotherapy can also reshape the tumor microenvironment by reducing immunosuppressive cell populations. Several agents selectively deplete or inhibit regulatory T cells (Tregs) and myeloid-derived suppressor cells, relieving suppressive pressure on effector T cells. This shift toward a more permissive immune milieu increases the likelihood that newly exposed or therapy-induced antigens will elicit productive T-cell responses. Simultaneously, chemotherapy promotes dendritic cell maturation and upregulates antigen-processing and presentation machinery within tumor cells, further enhancing antigen visibility.

Another crucial aspect of chemotherapy-induced immune modulation is the upregulation of inflammatory cytokine signaling, including type I interferons and IL-1 family mediators, which amplifies innate and adaptive immune activation. These cytokine changes may synergize with radiation-induced STING activation to enhance antigen presentation and T-cell recruitment [20].

Furthermore, targeted agents, most notably anti-VEGF therapies such as bevacizumab, contribute to immune remodeling via vascular normalization. By reducing abnormal, hypoxic vasculature and stabilizing the endothelial barrier, anti-VEGF therapy enhances T-cell trafficking and increases the infiltration of immune effector cells into the tumor. This vascular normalization overcomes a major physical barrier to immune engagement in colorectal tumors and has been shown to potentiate responses to ICIs across multiple cancer types.

Collectively, these mechanisms redefine chemotherapy not merely as a cytotoxic treatment component but as an essential immunomodulatory therapy that enhances antigen exposure, mitigates immunosuppression, and facilitates T-cell infiltration into the tumor. In the broader context of antigen remodeling, chemotherapy plays a pivotal role in priming CRC for a more effective response to immunotherapy [22].

**Key Take-Home Point:** The immunologic impact of chemotherapy varies by agent and includes immunogenic cell death, suppression of regulatory populations, and improved antigen presentation.

## 7. Epigenetic and Epitranscriptomic Alterations and RNA Editing as Sources of Neoepitope Diversity

Beyond genetic mutations, tumors can generate neoepitopes via a broad spectrum of epigenetic and epitranscriptomic alterations. Operating independently of DNA sequence changes, these mechanisms add a layer of antigenic diversity that is increasingly recognized as relevant to immunotherapy responses—particularly in cancers such as mismatch repair–proficient colorectal cancer (MSS CRC), where mutational neoantigens are limited.

One such mechanism is adenosine-to-inosine (A-to-I) RNA editing, mediated by ADAR enzymes [31]. RNA editing can alter codons, create recoding events, and generate noncanonical peptides that enter the immunopeptidome despite the absence of corresponding DNA mutations [7,26]. These edited peptides may be presented exclusively by MHC class I molecules, thus expanding the repertoire of potential T-cell targets. Although RNA editing has traditionally been studied in the context of innate immune suppression [32,33], its potential to generate neoepitope diversity is now an emerging area of interest in solid tumors. Notably, accumulating evidence indicates that chemoradiotherapy-induced cellular stress and interferon signaling can enhance RNA-editing activity, generating edited peptide variants with neoantigenic potential and thereby contributing to therapy-induced neoepitope diversity [7,26].

Similarly, aberrant RNA splicing—including exon skipping, intron retention, and aberrant exon inclusion—produces a wide variety of alternative protein isoforms [8,34,35]. Many of these isoforms contain frame-shifted or novel junctional peptides not found in the canonical proteome. Splicing dysregulation is common in CRC and may be exacerbated by cellular stress. Consequently, these peptides enrich the antigenic landscape and can serve as targets for T-cell recognition.

Translational noise is another key contributor to antigen diversity, encompassing errors in ribosomal decoding, non-AUG translation initiation, and altered proteasomal cleavage. These perturbations produce short-lived or unstable peptides that can still be processed and presented on MHC class I molecules. Concurrently, changes in proteasome activity and specificity—often observed under stress conditions—alter the peptide pool available for immune surveillance.

Chemoradiotherapy-induced cytotoxic stress further amplifies these epitranscriptomic perturbations. DNA damage responses influence RNA stability, splicing fidelity, and editing activity, while interferon signaling and oxidative stress reshape the transcriptome and proteome [26]. Collectively, these stress-induced processes generate epigenetic and epitranscriptomic plasticity, rendering the immunopeptidome more diverse and dynamic.

It is important to highlight that this phenomenon introduces a new conceptual framework for understanding MSS CRC: even tumors with a low mutational burden can achieve significant antigen diversification through epigenetic/epitranscriptomic and RNA-level remodeling.

This remodeling provides a mechanistic basis for sensitizing MSS tumors to immune checkpoint blockade by chemoradiotherapy, complementing DNA damage-derived neoantigens and helping convert immunologically cold tumors into more responsive ones.

**Key Take-Home Point:** Epigenetic and epitranscriptomic alterations, including RNA editing, may diversify the immunopeptidome and contribute to immune conversion in selected contexts.

## 8. Clinical Evidence: Combination of Cytotoxic Therapy and Immunotherapy

### 8.1. Long-Course Chemoradiotherapy (LCCRT) + ICI

Long-course chemoradiotherapy (LCCRT) has long been the standard neoadjuvant treatment for locally advanced rectal cancer, but recent studies have demonstrated that it may also serve as an effective immune primer to enhance the efficacy of ICIs [36,37,38]. The emerging paradigm suggests that cytotoxic therapy induces antigen remodeling—via DNA damage, transcriptional perturbations, and increased antigen presentation—thereby creating a tumor microenvironment that is more susceptible to PD-1 blockade. Several key clinical trials illustrate this concept. Major clinical trials evaluating the combination of chemoradiotherapy and immune checkpoint blockade in rectal cancer are summarized in Table 2.

#### 8.1.1. VOLTAGE Trial (EPOC1504)

The VOLTAGE trial was among the first to directly integrate ICI into LCCRT for resectable rectal cancer [36,37]. Following standard LCCRT (50.4 Gy with concurrent capecitabine), patients received nivolumab prior to surgery.

In the MMR-proficient (MSS) cohort—a group traditionally unresponsive to immunotherapy—the trial showed pathological complete response (pCR) rates of approximately 30%, markedly higher than those observed in historical controls treated with LCCRT alone.

Translational analyses revealed increased CD8^+^ T-cell infiltration and PD-L1 upregulation following chemoradiotherapy, supporting the hypothesis that LCCRT remodels antigenicity and immune accessibility. Patients with higher baseline CD8/Treg ratios or post-CRT PD-L1 expression derived the greatest benefit, consistent with the antigen remodeling model.

#### 8.1.2. NECTAR and Related Studies

The NECTAR trial and similar studies evaluating the sequencing of LCCRT and ICIs reinforce these findings, reporting favorable pCR and major pathological response rates in both MSS and MSI-H rectal cancers [38]. These studies collectively demonstrate that LCCRT acts not only as a cytoreductive modality but also as a biological sensitizer, priming the immune microenvironment to respond to PD-1 blockade.

### 8.2. Short-Course Radiotherapy (SCRT) + Chemotherapy + ICI in Total Neoadjuvant Therapy (TNT)

Short-course radiotherapy (SCRT), typically administered as 5 Gy × 5 fractions, has emerged as a promising platform for combining cytotoxic and immunotherapeutic strategies within total neoadjuvant therapy (TNT) regimens. Unlike long-course chemoradiotherapy, SCRT induces acute DNA damage and immunogenic stress signals, which may drive antigen remodeling more potently. Short-course radiotherapy (5 × 5 Gy; total 25 Gy) is an established alternative to long-course chemoradiotherapy and may offer logistical advantages and acceptable tolerability while preserving immunogenic potential. Optimizing radiation fractionation schedules may therefore represent one approach to balancing efficacy and toxicity in multimodal strategies. When followed by systemic chemotherapy and immune checkpoint inhibition (ICI), SCRT-based TNT has yielded encouraging pathological responses in both mismatch repair–proficient (MSS) and mismatch repair–deficient (dMMR) rectal cancer.

#### 8.2.1. UNION Trial (Phase III)

The UNION trial, a pivotal phase III study, directly compared SCRT + CAPOX + camrelizumab with conventional long-course chemoradiotherapy [40]. The TNT regimen achieved significantly higher pCR rates, validating SCRT-based immunogenic priming in a phase III setting. Notably, the trial demonstrated benefit across all biomarker subgroups, including MSS tumors, underscoring the broad applicability of SCRT + ICI combinations.

#### 8.2.2. Implications of SCRT-Based TNT for Immune Conversion

SCRT induces an intense, short-lived burst of DNA damage and inflammatory signaling, which may accelerate the formation of de novo neoantigens and enhance the visibility of cryptic antigens. Subsequent chemotherapy depletes immunosuppressive cell populations, while ICIs leverage the newly remodeled antigenic landscape.

This sequence reflects a biologically coherent framework in which SCRT initiates antigen remodeling, chemotherapy amplifies it, and ICI converts these changes into functional immune responses.

However, as summarized in Table 3, systemic treatment strategies combining chemotherapy with immune checkpoint inhibitors in metastatic colorectal cancer have thus far achieved limited rates of complete or deep responses when compared with chemoradiotherapy-based approaches. These clinical observations suggest that radiotherapy remains a critical component for effective antigen remodeling and immune sensitization, beyond what can be achieved by chemotherapy and immunotherapy alone.

**Key Take-Home Point:** Clinical trials suggest that combining cytotoxic therapy with ICIs may sensitize selected MSS rectal cancers, whereas dMMR tumors may respond to ICIs alone.

## 9. Biomarkers of Response to Antigen Remodeling and ICIs

With the increasing prominence of radiotherapy, chemotherapy, and immunotherapy in CRC, identifying biomarkers that predict responses to antigen remodeling and subsequent immune activation is critical. These biomarkers not only guide patient selection but also provide mechanistic insights into how cytotoxic therapies enhance the response to immunotherapy. Several classes of biomarkers—covering immune infiltration, antigen load, transcriptomic signatures, and features of the tumor microenvironment—have been studied in this context.

### 9.1. CD8^+^ T-Cell Infiltration and the CD8/Treg Ratio

One of the strongest predictors of response to PD-1 blockade following chemoradiotherapy is the density of CD8^+^ cytotoxic T cells within the tumor and, more specifically, the CD8/Treg ratio. In trials such as VOLTAGE, patients with higher pretreatment CD8/Treg ratios showed markedly higher pathological complete response rates after LCCRT followed by nivolumab [10,36,37]. This biomarker reflects a microenvironment poised for immune activation, where antigen remodeling can further enhance T-cell priming.

### 9.2. PD-L1 Expression and Adaptive Immune Activation

Although PD-L1 expression is heterogeneous in CRC and is not a standalone predictor of ICI efficacy, its upregulation following radiotherapy or chemoradiation has been consistently associated with improved outcomes in combination strategies [36,37]. This posttreatment induction of PD-L1 represents a form of adaptive immune resistance, signaling the concurrent activation of interferon pathways and T-cell engagement—both hallmarks of successful antigenic remodeling.

### 9.3. Tumor Mutational Burden (TMB) and Clonal Neoantigen Load

Baseline TMB is typically low in mismatch repair–proficient (MSS) tumors; however, radiation-induced DNA damage and chemotherapy-induced stress can transiently increase neoantigenic complexity [27]. Clinical observations suggest that patients whose tumors exhibit a higher clonal neoantigen burden or increased mutational activity after LCCRT are more likely to benefit from ICIs. In contrast, mismatch repair–deficient (dMMR) tumors have intrinsically high TMB, explaining their exceptional sensitivity to ICI monotherapy [3,16].

### 9.4. Consensus Molecular Subtype Classification

Transcriptomic subtypes correlate with immune architecture and treatment response. CMS1 (immune-active) and CMS3 (metabolic/immune-intermediate) tumors exhibit greater responsiveness to CRT + ICI regimens. CMS4, characterized by stromal activation and TGF-β–driven immune exclusion, often exhibits poorer responses, underscoring the role of stromal barriers in limiting the impact of antigen remodeling [10,12,13].

### 9.5. RNA Editing and Epigenetic/Epitranscriptomic Signatures

In addition to DNA mutations, epitranscriptomic alterations, including RNA editing and splicing-derived peptide variants, may serve as biomarkers of antigenic diversity. Tumors with higher baseline RNA-editing activity or stress-induced transcriptomic diversity may generate noncanonical neoepitopes during cytotoxic therapy, thereby providing a mechanistic basis for response even in MSS tumors with a low DNA-level neoantigen load [7,26,31].

### 9.6. Microbiome-Associated Biomarkers

Emerging evidence links gut microbiome composition to ICI responsiveness. The presence of immunostimulatory taxa and the absence of suppressive species, such as Fusobacterium nucleatum, may enhance dendritic cell activation and T-cell priming following CRT [48,49,50]. Given that antigen processing relies on effective communication between APCs and T cells, microbiome-associated immune competence may influence the success of multimodal regimens.

Collectively, these biomarkers highlight a consistent theme: successful immunotherapy in CRC requires both antigen abundance and a receptive immune microenvironment. While cytotoxic therapies can enhance antigenicity, the capacity of T cells to recognize and respond to these modified antigens depends on both preexisting and inducible immune characteristics, as reflected by these biomarkers.

**Key Take-Home Point:** No single biomarker currently predicts response to antigen remodeling strategies in colorectal cancer; integrated genomic, transcriptomic, and microenvironmental profiling will likely be required for effective patient selection.

## 10. Future Directions: Toward Multimodal Immune Conversion Strategies

Rapid advances in genomics and epigenomics are reshaping our understanding of CRC in the context of tumor immunity. Technologies such as spatial transcriptomics, single-cell sequencing, and AI-assisted neoantigen prediction increasingly suggest that the distinction between cold and hot tumors is dynamic rather than fixed. As radiotherapy, chemotherapy, and stress-induced transcriptomic alterations reveal new layers of antigenic diversity, we can envision a future where the immune phenotype of CRC is deliberately reprogrammed rather than merely observed. Prospective longitudinal sampling integrating immunopeptidomics, TCR sequencing, and spatial immune profiling will be required to validate antigen remodeling. A minimal biomarker panel should include antigen presentation integrity, interferon signatures, and T-cell clonality (Table 4).

Genomic instability, RNA modifications, and epigenetic/epitranscriptomic plasticity contribute to a dynamic antigenic landscape that may be more malleable than previously thought. Integrating these insights with AI-driven modeling could enable clinicians to predict tumor responses to cytotoxic therapy, identify the most favorable timing for antigen presentation, and determine the best way to schedule or tailor immunotherapy. Spatial profiling will further elucidate sites of immune exclusion and how to target specific microregions to achieve more uniform immune activation.

Whether specific MSS colorectal cancers are intrinsically resistant to therapy-induced antigen remodeling remains unclear. Defining molecular and immunologic features that predict resistance to antigen diversification will be a critical priority for future translational research. In addition, the potential emergence of antigen-loss variants and T-cell exhaustion following therapy may limit the durability of immune conversion. Longitudinal studies integrating antigen presentation profiling and functional T-cell assessment will be required to clarify these dynamics. Comprehensive paired pre- and post-therapy analyses—including immunopeptidomics, TCR repertoire profiling, antigen presentation machinery assessment, interferon signatures, and spatial immune mapping—remain limited in colorectal cancer. The absence of such longitudinal data represents a major translational gap that constrains the mechanistic interpretation of clinical responses. Targeting immunosuppressive pathways, including TGF-β signaling and myeloid cell-mediated suppression, may represent complementary strategies to enhance the durability of antigen remodeling. Further translational and clinical studies will be required to determine whether modulation of inflammatory signaling pathways, such as JAK inhibition, can overcome resistance in selected patients [33].

**Figure 1:**
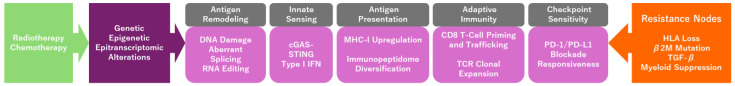
Antigen Remodeling and Immunity in Colorectal Cancer Treatment. Future validation of antigen remodeling will require prospective longitudinal sampling incorporating immunopeptidomics, TCR repertoire sequencing, and spatial immune profiling. A minimal translational biomarker panel may include assessment of antigen presentation machinery, interferon signaling signatures, T-cell clonality, and spatial CD8^+^ infiltration. Such integrative approaches will be essential to mechanistically link antigen diversification to clinical response.

Future validation of antigen remodeling will require prospective longitudinal sampling incorporating immunopeptidomics, TCR repertoire sequencing, and spatial immune profiling. A minimal translational biomarker panel may include assessment of antigen presentation machinery, interferon signaling signatures, T-cell clonality, and spatial CD8^+^ infiltration. Such integrative approaches will be essential to mechanistically link antigen diversification to clinical response (Figure 1).

In this emerging framework, CRC is no longer defined solely by its intrinsic lack of immunogenicity but by its potential for engineered immune conversion. As our ability to analyze and manipulate the antigenic landscape improves, the long-standing dichotomy between cold and hot tumors may give way to a more nuanced, programmable approach to optimizing immunotherapy. 

**Key Take-Home Point:** Defining predictive biomarkers and validating longitudinal antigen remodeling through integrative multi-omics will be essential for future clinical translation.

## 11. Conclusions

CRC has traditionally been characterized as immunologically cold, yet emerging evidence shows that this state is neither fixed nor inevitable. Radiotherapy, chemotherapy, and epigenetic/epitranscriptomic alterations can collectively remodel tumor antigenicity, paving the way for significant immunotherapy responses in tumors previously considered refractory. Understanding and harnessing this antigen-remodeling capacity will be pivotal to the next generation of treatment strategies. As analytic tools advance and therapeutic combinations evolve, the boundary between cold and hot tumors may become less of a biological limitation and more of a modifiable therapeutic parameter.

## Figures and Tables

**Table 1:** Mechanisms of therapy-induced neoantigen generation classified by genetic versus epigenetic/epitranscriptomic origin.

Mechanism/Change	Therapy	Class (Genetic/Epigenetic)	Neoantigen/Antigen Source	Key Immunologic Consequence	Refs
DNA double-strand breaks (DSBs) → misrepair → new mutations	Radiotherapy	Genetic	De novo mutation–derived neoantigens (missense/indel/frameshift peptides)	Expands mutational/neoepitope space (esp. in surviving clones)	[18,19]
Radiation alters antigen processing & MHC-I peptide repertoire	Radiotherapy	Epigenetic/functional	“Remodeled” immunopeptidome (newly presented peptides)	Increases MHC-I expression and diversifies presented peptides	[5]
cGAS–STING activation via cytosolic DNA from irradiated tumor	Radiotherapy	Epigenetic/innate sensing	Not a neoantigen per se; promotes antigen priming context	Type I IFN program, DC priming, synergy with ICI	[20,21]
Immunogenic cell death (ICD)	Chemotherapy (subset-dependent)	Epigenetic/immunologic	Release/exposure of tumor antigens (incl. neoantigens) with danger signals	Calreticulin exposure, ATP/HMGB1 signaling → DC activation	[6]
Immune suppressor cell modulation and vascular normalization	Chemotherapy/Targeted therapy	Epigenetic/Microenvironmental	Enhanced antigen presentation and immune cell infiltration	Cytotoxic chemotherapy and anti-VEGF therapy reduce immunosuppressive cells and promote vascular normalization, thereby enhancing antigen presentation and effector T-cell infiltration	[6,22,23]
Alternative splicing dysregulation → neojunction peptides	Stress/therapy (incl. chemoRT)	Epigenetic/transcriptomic	Splicing-derived neoantigens (neojunction peptides)	Creates “noncanonical” neoepitopes even in low-TMB tumors	[8,24]
RNA editing (A-to-I) → recoded peptides	Stress/therapy; intrinsic RNA editing	Epigenetic/epitranscriptomic	RNA editing–derived neoepitopes (recoding)	Adds postgenomic antigen diversity; potential T cell targets	[7]
DNA demethylation → endogenous retroelement re-expression (“viral mimicry”)	Epigenetic drugs (DNMTi; conceptually synergistic with chemoRT/ICI)	Epigenetic	ERV/dsRNA-driven immunogenic transcripts (antigenic + IFN context)	Type I IFN signaling; improved immune visibility	[25]

DSB, DNA double-strand break; NHEJ, nonhomologous end joining; MHC-I, major histocompatibility complex class I; cGAS, cyclic GMP–AMP synthase; STING, stimulator of interferon genes; IFN, interferon; DC, dendritic cell; ICD, immunogenic cell death; VEGF, vascular endothelial growth factor; TME, tumor microenvironment; RNA, ribonucleic acid; A-to-I, adenosine-to-inosine; ERV, endogenous retrovirus; DNMTi, DNA methyltransferase inhibitor.

**Table 2:** Clinical trials combining radiotherapy/chemoradiotherapy with immune checkpoint inhibitors in rectal cancer.

Trial/Regimen	Population	Design	Key Outcomes (More Detailed)	Notes (Why It Matters for “Antigen Remodeling”)	Refs
VOLTAGE (CRT → nivolumab → surgery)	LARC, MSS + exploratory MSI-H	Phase I/II, sequential consolidation ICI	Pathological complete response (pCR) rate of 30% in MSS tumors, markedly exceeding historical controls (~10–15% with CRT alone). In exploratory MSI-H/dMMR cohort, pCR rate exceeded 60%. Grade ≥3 immune-related adverse events occurred in 3 of 39 MSS patients (7.7%), with no treatment-related deaths.	Classic “cold MSS rectal” setting where CRT priming + PD-1 blockade produces unexpectedly deep responses; links clinically to antigen remodeling concept	[36]
VOLTAGE 3-year outcomes	Same cohort follow-up	Long-term follow-up	3-year relapse-free survival (RFS): 79.5% in MSS tumors; 100% in MSI-H tumors. 3-year overall survival (OS): 97.4% in MSS tumors; 100% in MSI-H tumors. Sustained local control with no excess late toxicity attributable to nivolumab consolidation.	Shows that the short-term biological synergy can translate into durable disease control; biomarker hints (PD-L1, CD8/eTreg, etc.) help frame “who becomes hot”	[37]
VOLTAGE cohort D (CRT → nivolumab + ipilimumab)	LARC	Conference abstract/full-text proceedings	Feasible administration of dual immune checkpoint blockade after CRT. Reported high rates of major pathological response, with manageable immune-related toxicity. Early efficacy signals suggest enhanced immune activation compared with PD-1 monotherapy consolidation.	Mechanistically “more ignition”: CRT reshapes antigen presentation; dual ICI amplifies priming/expansion	[39]
NECTAR (PD-1 blockade + long-course chemoradiotherapy)	LARC (multi-center)	Phase II	Pathological complete response rate of approximately 25–30%. Grade ≥3 treatment-related adverse events were observed in 2 of 50 patients (4.0%), including grade 3 immune-related colitis and rash. Translational analyses demonstrated increased intratumoral CD8^+^ T-cell infiltration posttreatment.	Supports that LCCRT itself can be an antigen-remodeling platform and that adding PD-1 blockade can exploit it	[38]
UNION (SCRT → CAPOX + camrelizumab vs. LCRT → CAPOX)	LARC, randomized	Phase III	pCR rate: 39.8% in SCRT–CAPOX–camrelizumab arm vs. 15.3% in control arm. No significant increase in postoperative complications or grade ≥3 adverse events between arms. Benefit observed across biomarker subgroups, including MSS tumors.	Strong randomized evidence that SCRT-based TNT + PD-1 can markedly raise pCR; clinically anchors “cold → responsive” framework	[40]

LARC, locally advanced rectal cancer; CRT, chemoradiotherapy; LCCRT, long-course chemoradiotherapy; SCRT, short-course radiotherapy; ICI, immune checkpoint inhibitor; PD-1, programmed cell death protein 1; CTLA-4, cytotoxic T-lymphocyte-associated protein 4; pCR, pathological complete response; RFS, relapse-free survival; OS, overall survival; MSS, microsatellite stable; MSI-H, microsatellite instability-high; dMMR, deficient mismatch repair; TNT, total neoadjuvant therapy; Gy, gray.

**Table 3:** Systemic therapy combined with immune checkpoint inhibition in metastatic colorectal cancer.

Strategy/Trial	Line/Population	Design	Key Outcomes (More Detailed)	Interpretation for Antigen Remodeling	Refs
CheckMate 9X8 (mFOLFOX6 + bevacizumab ± nivolumab)	first-line mCRC (predominantly MSS/pMMR)	Randomized phase II	Median progression-free survival (PFS): ~11.9 months with nivolumab arm vs. ~11.3 months control (not meeting primary endpoint). Higher objective response rates and longer duration of response in selected subgroups. Safety profile consistent with chemotherapy plus bevacizumab.	A reality-check: “add PD-1 to chemo” is not automatically antigen remodeling—context (metastatic site, biology, schedule) matters	[41]
AtezoTRIBE (FOLFOXIRI + bevacizumab ± atezolizumab)	first-line mCRC	Randomized phase II	Median PFS improved from 11.5 months (control) to 13.1 months with atezolizumab. Objective response rate exceeded 60% in both arms. Acceptable safety with no unexpected immune-related toxicities.	Triplet chemo + VEGF blockade may improve immune infiltration; sets up discussion of vascular normalization + antigen release	[42]
KEYNOTE-651 (pembrolizumab + binimetinib ± chemotherapy)	MSS/pMMR mCRC	Phase Ib cohorts	Objective response rates ranged from 10 to 25%, depending on cohort and regimen. Median PFS generally <6 months across cohorts. Toxicity manageable; immune-related AEs consistent with PD-1 blockade.	“MEK/chemo + PD-1” tries to rewire immune context; useful to contrast with chemoRT where local priming is stronger	[43,44]
REGONIVO (regorafenib + nivolumab)	Heavily pretreated GC/CRC (mostly MSS/pMMR)	Phase Ib	Objective response rate in CRC cohort approximately 33%. Median PFS around 7–8 months. Responses enriched in patients without liver metastases.	Anti-angiogenic + multikinase “microenvironment editing” may unlock ICI in subsets; supports “TME remodeling” axis	[45]
Regorafenib + nivolumab (Western MSS/pMMR mCRC)	MSS/pMMR mCRC	Phase II	Objective response rate approximately 7–10%. Median PFS ~3–4 months. Liver metastasis identified as a dominant resistance factor.	Helps discuss why “same combo” behaves differently by cohort/setting (microbiome, liver metastasis, immune exclusion)	[46]
RIN (regorafenib + ipilimumab + nivolumab)	Refractory MSS mCRC	Phase I	Objective response rate approximately 20–30% in dose-optimized cohorts. Median PFS ~5–6 months. Immune-related grade ≥3 adverse events manageable with protocol-defined dose adjustments.	Illustrates escalation approach: if antigen remodeling is partial, intensify T cell priming (CTLA-4) + relieve suppression	[47]

mCRC, metastatic colorectal cancer; pMMR, mismatch repair–proficient; ORR, objective response rate; PFS, progression-free survival; OS, overall survival; VEGF, vascular endothelial growth factor; MEK, mitogen-activated protein kinase kinase; AE, adverse event; GC, gastric cancer.

**Table 4:** Proposed Experimental Framework to Validate Antigen Remodeling in Colorectal Cancer.

Objective	Methodology	Sample Type	Readout	Clinical Relevance
Detect therapy-induced neoepitopes	Immunopeptidomics (LC–MS/MS)	Pre/post tumor biopsies	MHC-bound peptide repertoire changes	Direct evidence of neoantigen diversification
Assess T-cell recognition	Functional T-cell assays (ELISPOT, tetramer staining)	Tumor-infiltrating lymphocytes	Neoepitope-specific T-cell activation	Proof of immunogenicity
Evaluate clonal dynamics	TCR sequencing	Tumor and peripheral blood	TCR expansion and clonality	Immune selection pressure
Characterize spatial immune remodeling	Spatial transcriptomics/multiplex IHC	Tumor tissue	Immune cell proximity and density	Immune conversion assessment
Monitor systemic response	Circulating tumor DNA/cytokine profiling	Plasma samples	Dynamic immune biomarkers	Non-invasive monitoring

## Data Availability

All data is included in the manuscript.

## Abbreviations

The following abbreviations are used in this manuscript:

A-to-I	adenosine-to-inosine
AE	adverse event
CAF	cancer-associated fibroblast
cGAS	cyclic GMP–AMP synthase
CRC	colorectal cancer
CRT	chemoradiotherapy
DC	dendritic cell
dMMR	mismatch repair-deficient
DSB	DNA double-strand break
ERV	endogenous retrovirus
Gy	gray
ICD	immunogenic cell death
ICI	immune checkpoint inhibitor
IFN	interferon
LARC	locally advanced rectal cancer
LCCRT	long-course chemoradiotherapy
MDSC	myeloid-derived suppressor cell
mCRC	metastatic colorectal cancer
MHC-I	major histocompatibility complex class I
MSI-H	microsatellite instability–high
MSS	microsatellite stable
NHEJ	non-homologous end joining
ORR	objective response rate
OS	overall survival
pCR	pathological complete response
pMMR	mismatch repair–proficient
PFS	progression-free survival
RNA	ribonucleic acid
SCRT	short-course radiotherapy
STING	stimulator of interferon genes
TME	tumor microenvironment
TNT	total neoadjuvant therapy
Treg	regulatory T cell
VEGF	vascular endothelial growth factor
